# Paranasal Rosai-Dorfman Disease with Osseous Destruction

**DOI:** 10.1155/2017/1453097

**Published:** 2017-02-21

**Authors:** Kevin Hur, Changxing Liu, Jeffrey A. Koempel

**Affiliations:** ^1^Caruso Department of Otolaryngology, Keck School of Medicine, University of Southern California, Los Angeles, CA 90033, USA; ^2^Division of Otolaryngology, Head and Neck Surgery, Children's Hospital Los Angeles, Los Angeles, CA 90027, USA

## Abstract

Rosai-Dorfman disease is a rare histiocytic proliferative disorder of unknown etiology typically characterized by cervical lymphadenopathy. Extranodal involvement often manifests in the head and neck region. We present a 10-year-old male who presented to our hospital with left epiphora from an aggressive paranasal mass invading the left orbit with osseous destruction. The mass was surgically biopsied and debulked with histopathological examination revealing Rosai-Dorfman disease. Although rarely found in the sinuses, Rosai-Dorfman disease should be considered when evaluating sinonasal masses.

## 1. Introduction

Rosai-Dorfman disease, first described in 1969, is a nonneoplastic histiocytic proliferative disorder of unknown etiology and pathogenesis [1]. The most common presentation is painless bilateral cervical lymphadenopathy accompanied by fever, leukocytosis, increased erythrocyte sedimentation rate, and hypergammaglobulinemia [2]. However, patients with extranodal disease oftentimes lack any constitutional symptoms [2].

Extranodal disease has been reported in up to 43% of cases and commonly involved sites include the skin, central nervous system, orbit and eyelid, upper respiratory tract, and gastrointestinal tract [2–6]. The disease has a variable clinical presentation and requires a pathological review for definitive diagnosis, which is characterized by massive sinusoidal dilation that contains histiocytes, lymphocytes, and plasma cells. Emperipolesis within the histiocyte cytoplasm is a pathognomonic finding [2–4].

Oftentimes patients do not require treatment as the disease has a self-limiting course. Nevertheless, surgical resection is recommended for symptomatic disease. We herein describe a rare case of extranodal sinonasal Rosai-Dorfman disease with osseous destruction of the orbit.

## 2. Case Report

**Figure 1:**
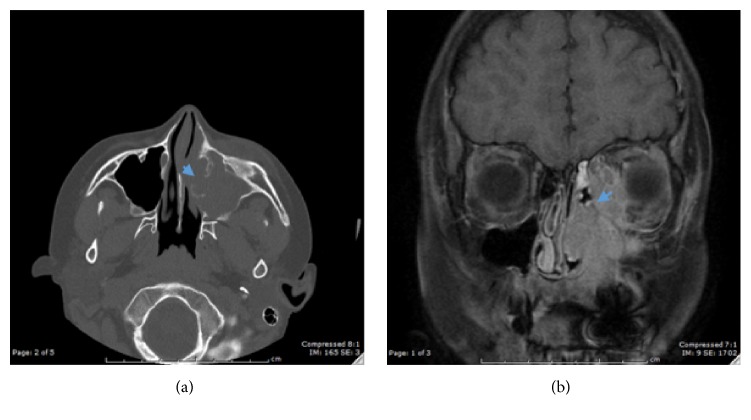
Image of mass in left anterior ethmoid cell extending into left medial aspect of orbit on axial sinus CT without contrast (a), and MRI orbit T1 after contrast (b). Blue arrow indicates area of bony destruction.

**Figure 2:**
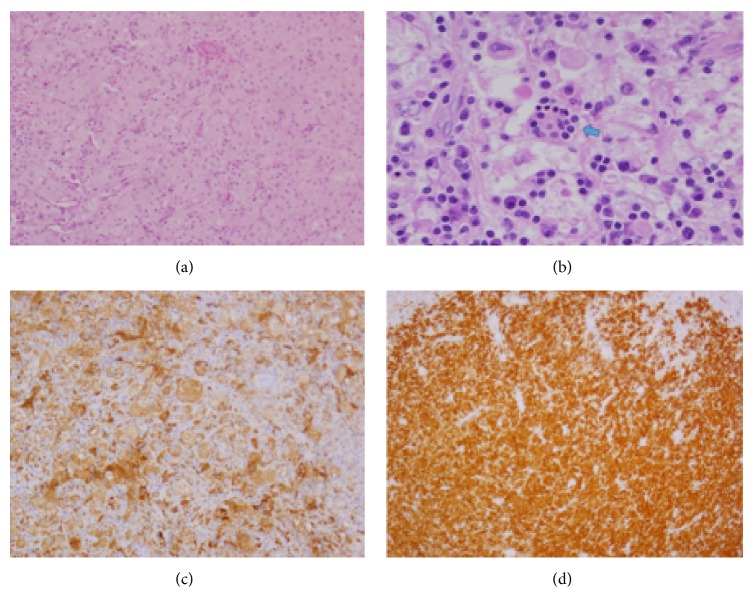
Permanent sections showing histology of Rosai-Dorfman disease; low power 100x (a) and high power 400x (b) hematoxylin and eosin stain showing histiocytic infiltrate. Blue arrow indicates emperipolesis. Positive immunostaining for CD68 (c) and S100 (d).

A 10-year-old Hispanic male presented to the emergency room at Children's Hospital Los Angeles after 6 months of left eye tearing. He was seen by an ophthalmologist a month earlier who found left nasolacrimal duct obstruction on exam. A CT orbit revealed a soft tissue mass involving the medial aspect of the left ethmoid and maxillary sinus with osseous destruction (see Figure 1). The patient denied vision changes, weight loss, fevers, chills, or other symptoms. Laboratory findings were within normal limits with no leukocytosis, increased erythrocyte sedimentation rate, or hypergammaglobulinemia. The mass was not visualized on nasal endoscopy due to a narrow nasal cavity and enlarged middle turbinate. Therefore, an orbital biopsy by ophthalmology was performed, revealing lymphoid tissue with histiocyte proliferation. However, due to the aggressive symptomatic clinical presentation of the sinonasal orbital mass, the patient underwent a left orbitotomy for debulking of the mass. The histopathological examination revealed sheets of histiocytes mixed with few lymphocytes and plasma cells consistent with Rosai-Dorfman disease. There was phagocytic activity identified in the sinus histiocytes characterized as emperipolesis. The histiocytes were CD68(+), S-100(+), C1Da(−), Desmin(−), and EBV(−) (see Figure 2). At 3-month follow-up, the patient reported resolution of left eye tearing and was asymptomatic. Therefore, no further postoperative treatment or imaging was obtained.

## 3. Discussion

Rosai-Dorfman disease (RDD) presents typically in childhood or early adulthood with a higher incidence in males and African-Americans [2]. The etiology of RDD is currently unknown and considered idiopathic. Proposed etiologies include chronic infection or immune dysfunction leading to an exaggerated response to viral agents such as the Epstein-Barr virus, but the overall evidence does not support any one specific theory [2, 4].

When there is an extranodal presentation of RDD, the head and neck region is frequently involved. However, the presentation is variable with case reports of RDD identified in the trachea, nasal septum, dura, orbit, parotid, and so forth [4–14]. The paranasal sinuses are the most common extranodal site of involvement after skin, followed by the orbit, bone, salivary gland, and central nervous system [4]. Patients often present with nasal obstruction, epistaxis, hyposmia, or anosmia [4, 8–11].

A review of RDD imaging manifestations in the head and neck at one institution over a 10-year period found 5 out of 13 head and neck RDD cases had extranodal disease in the paranasal sinuses. None of the paranasal RDD had osseous destruction on imaging [8]. However, there is one case report of sinonasal RDD with osseous destruction of the premaxilla [12]. Also, a pathology quiz case described a young girl with sinonasal RDD extending into the right orbit, but whether the osseous destruction was from her prior surgeries or the disease was unclear [14]. Otherwise, there were no reported cases of osseous destruction of the bony orbit from paranasal RDD identified from our literature search.

The diagnostic workup for RDD relies on histopathologic examination of an incisional or excisional biopsy [2, 11]. The differential diagnosis is extensive and includes other histiocytic proliferative diseases including lymphoma, tuberculosis, granulomatosis with polyangiitis, sarcoidosis, and Langerhans cell histiocytosis. Malignant etiologies such as nasopharyngeal carcinoma and lymphoma should also be considered, especially if aggressive features are seen radiologically as seen in this patient. Identification of emperipolesis within the histiocyte cytoplasm is pathognomonic as originally described by Rosai et al., though histiocytes are less common in extranodal tissue [1–7, 15]. In cases where few histiocytes with emperipolesis are seen, useful immunohistologic markers for RDD include the S100 protein, which is a marker for dendritic cells in lymph nodes and Langerhans cells in skin, and CD68, a pan-macrophage antigen (see Figure 2) [3, 4].

RDD generally has a benign course and does not require treatment. In one review, 82% of untreated patients show spontaneous regression over a period of weeks to years without treatment [16, 17]. However, massive lymphadenopathy, involvement of multiple organs, immunologic abnormalities, and anemia can lead to poor prognosis and in very few cases may be fatal [3, 4]. If patients are symptomatic, the standard treatment is surgical resection. Systemic treatment for extensive disease has not been well established due to rarity of the disease though reported treatment modalities such as steroids, radiation, and chemotherapy have had varying success based on a small case series [2–7]. In patients that require systemic treatment due to symptomatic extensive disease, steroids are typically the first treatment option, having been shown to produce responses in classical and extranodal RDD [2–7]. Radiotherapy can preserve vital organ function in orbital, airway, or CNS involvement but there are no standardized guidelines for treatment in the literature. If steroids and radiotherapy are unsuccessful, chemotherapy can be considered, though data is limited on its effectiveness [2–7].

After diagnosis or treatment, Dalia and colleagues recommend surveillance with clinical and laboratory testing performed every 3–6 months for the first 2 years and then yearly afterwards [2]. Contrast CT scans can be obtained if patients develop symptoms or there is concern for relapse [2].

## 4. Conclusion

Rosai-Dorfman disease is a rare entity with variable presentations in the head and neck region. Although uncommon, Rosai-Dorfman disease should be considered in the differential diagnosis when evaluating sinonasal masses in children.
